# A pragmatic approach to teaching physician assistant students basic pain management

**DOI:** 10.17305/bb.2024.11109

**Published:** 2024-08-31

**Authors:** Chelsey Hoffmann, Ryan S D’Souza

**Affiliations:** 1 Department of Anesthesiology and Perioperative Medicine, Mayo Clinic, Rochester, MN, USA

**Keywords:** Pain, pain management, opioids, active learning, framework

## Abstract

Chronic pain is increasing in prevalence, with new cases now outnumbering those of diabetes, depression, or hypertension. Advanced practice providers have reported that their training in pain management inadequately prepared them to care for patients suffering from painful conditions. In response, the authors of this work developed a basic pain management conceptual framework to provide physician assistant (PA) students with the foundational knowledge necessary to manage and treat patients suffering from a wide variety of painful conditions. The devised framework activity includes categories of pain management, such as conservative therapies, medications, injections, minimally invasive procedures, and moderate to highly invasive procedures. This framework can be incorporated into the existing PA educational curriculum and presented alongside a realistic pain patient case study. Furthermore, other health science educational programs, such as nurse practitioner or pharmacy programs, could adopt this framework to increase student knowledge in pain management.

## Introduction

According to the Centers for Disease Control and Prevention (CDC), an estimated 20.9% of U.S. adults (51.6 million individuals) experienced chronic pain in 2021 [1]. Additionally, the National Institutes of Health (NIH) recently found that new cases of chronic pain are occurring more frequently among U.S. adults than new cases of diabetes, depression, or hypertension [2]. Alongside concerns regarding the increasing prevalence of chronic pain is the ongoing public health issue of opioid abuse, misuse, and diversion. Recently published statistics showed that over 16 million people worldwide suffer from opioid use disorder (ranging from dependence to addiction), including over 2.1 million individuals in the United States [3]. Furthermore, in October 2017, the U.S. federal government declared the opioid epidemic a public health emergency, and new research suggests we are entering the third decade of this crisis [4, 5].

At odds with these findings, published research has shown that “14% of physician assistant/associate (PA) program directors report that pain management is not an entry-level PA competency” [6]. Given the increasing prevalence of painful conditions and the ongoing opioid epidemic, PA programs must incorporate education on basic pain management into their curricula. This sentiment was also expressed by researchers in 2019 who published findings on the current state of pain management curriculum within PA education [6]. Fortunately, their study revealed that only 14% of participating PA programs reported that pain management was not included in their curricula [6]. The reasons cited for excluding pain management included insufficient time (39%) and the lack of a mandate from accreditation bodies (32%) [6]. While these findings suggest that many PA programs do include pain management modules or activities within their existing curricula, other research has shown that healthcare providers working in community clinics report that their training for treating chronic painful conditions was less than adequate [7].

**Table 1:** Conceptual framework for basic pain management*

**Category**	**Options**
Conservative	–Transcutaneous electrical nerve stimulation (TENS), magnetic field therapy;–Topicals (ice or heat, icy-hot with lidocaine, asper cream with lidocaine, bengay, lidocaine patch);–Physical therapy, hydrotherapy, dry needling, desensitization, mirror-therapy;–Chiropractor, spinal traction, ultrasound, manual soft tissue therapy;–Acupuncture;–Massage;–Cognitive behavioral therapy, yoga, tai-chi, biofeedback.
Medications	–Opioids (morphine, oxycodone, hydrocodone, hydromorphone);–Non-opioids: over-the-counter (OTC) medications (acetaminophen, ibuprofen), anticonvulsants (gabapentin, pregabalin), antidepressants (duloxetine, amitriptyline, nortriptyline), muscle relaxants (cyclobenzaprine, tizanidine), and atypical analgesics (acetyl-l-carnitine, alpha lipoic acid).
Injections	–Corticosteroid +/− local anesthetic (epidural injection, nerve blocks, joint injections);–Platelet-rich-plasma (PRP);–Stem cell;–Viscosupplement (hyaluronic acid).
Minimally invasive procedures	–Ablative therapy (radiofrequency denervation, cryoablation, high-intensity focused ultrasound);–Neuromodulation (spinal cord stimulation, dorsal root ganglion stimulation, peripheral nerve stimulation);–Intrathecal drug delivery; –Minimally invasive lumbar decompression (MILD).
Moderate/highly invasive procedures	–Open spinal surgery +/− fusion;–Total joint replacement.

*Examples are provided throughout the table to aid the faculty members and students. However, this table is not all-encompassing. When utilized in classroom activities, students should be provided a copy of Table 1 with only row and column titles included.

Until a standardized basic pain management curriculum is written, approved, or mandated for incorporation into PA education, an interim solution is necessary to equip PA faculty and students with a conceptual framework for approaching patients suffering from pain. The primary aim of this manuscript is to describe and propose such a framework. This article does not, however, address the issue of identifying and managing opioid use disorders, as that topic is outside the scope of this manuscript.

PA students will undoubtedly encounter patients suffering from both acute and chronic pain during their clinical rotations. Once they become practicing PAs, they will be responsible for managing these patients in primary care settings and various medical subspecialties. As faculty, it is our duty to prepare students to the best of our ability.

## Timing and framework activity

The following proposed pain management conceptual framework activity may be most feasible to implement for students who have already completed their pharmacology courses. However, the timing of implementation can be adjusted according to the unique curriculum and course sequence of each PA program. Other opportunities for implementation may include courses, such as neurology or orthopedics.

In this framework activity, the faculty member acts as the facilitator while students actively participate in “completing” the framework. In other words, the faculty member provides the scaffolding, while students fill in the details. Faculty can step in to support learners if they encounter difficulties or challenges during the process.

First, faculty should explain to students the difference between acute and chronic pain. Acute pain is typically sudden in onset, intense, and of shorter duration, whereas chronic pain persists beyond three or six months, depending on the specific definition used by various pain society guidelines [7]. Second, when approaching patients suffering from pain and determining treatment options, students can utilize the framework provided in Table 1. The faculty member can either draw this framework on a whiteboard or provide hard copies of Table 1 with only the row and column headings included. Faculty should explain to students that the categories are organized by degree of risk, ranging from least to most risk. One of the benefits of this exercise is that students may think of therapeutic options that even faculty members may not have considered, providing a learning opportunity for both. Furthermore, this exercise can grow and evolve as technology and pain management options continue to advance.

It is beneficial to discuss with students or prompt them to consider the pros and cons, as well as the contraindications, of any treatment or medication they are considering for their patient suffering from pain. Furthermore, emphasis should be placed on a patient-centered approach to pain management. Providers should be able to discuss basic strategies or options for pain management with their patients but should be willing to adjust according to the patient’s expressed needs regarding their health.

If desired, faculty can also reference and utilize Table 1 after presenting a realistic pain patient case. For example, the history and physical exam of a patient suffering from acute-on-chronic low back pain can be provided first, with the students subsequently filling in reasonable management/treatment options in Table 1.

### Conservative treatment

Conservative treatment measures include prescriptions for a transcutaneous electrical nerve stimulator (TENS), topical agents, physical therapy, chiropractic care, acupuncture, massage, cognitive behavioral therapy (CBT), yoga, tai chi, or biofeedback. It is important to note that patients may resist prescriptions for physical therapy, stating that previous attempts exacerbated their pain. PA students must be prepared to handle these conversations and explain that initially, retraining, rehabilitating, and strengthening muscles can indeed result in short-term discomfort. However, the goals are long-term functional gains and pain relief.

### Medications

The options for the pharmacologic management of acute or chronic pain are numerous. To ease the burden of remembering a multitude of medication options for pain management, students should categorize medications into one of the two primary categories: opioids or non-opioids. Non-opioids can then be further subcategorized into groups, such as anticonvulsants, antidepressants, muscle relaxants, and other categories based on their primary mechanisms of action.

Depending on the patient’s painful condition and prior treatments attempted, over-the-counter (OTC) medications may be a good first option. It is crucial to ensure that patients are neither overdosing nor underdosing these medications. For example, patients may report not benefiting from acetaminophen (Tylenol) or standard non-steroidal anti-inflammatory drugs (NSAIDs), such as ibuprofen or naproxen. However, upon further conversation, the PA may discover that the patient was underdosing the medication. Conversely, care must be taken to ensure that the patient is not overdosing on OTC medications, as acetaminophen can lead to hepatotoxicity, and NSAIDs can cause a variety of side effects, including renal injury and gastrointestinal effects.

When discussing various pain medication options, faculty should take the opportunity to reinforce PA students’ knowledge from their pharmacology courses. Discussions can be held regarding drug metabolism, drug–drug interactions, potential side effects, and contraindications.

### Injections

Once a practicing PA (or PA student) has exhausted conservative treatments and one or more pharmacologic options, consideration may be given to offering injection or interventional therapies. In some practices, PAs are trained to perform ultrasound-guided or non-guided corticosteroid injections, while in other practices, these procedures are deferred to specialists in pain medicine, sports medicine, or orthopedics. Regardless, it is essential for PA faculty and students to understand the existing options available so that they never feel as though opioid medications are the only remaining option in their toolkit.

A variety of injectables are now available for treating painful conditions, some involving corticosteroids and others that do not. While a comprehensive review of all injectable options for pain management is outside the scope of this manuscript, it is worth noting that both PA students and faculty may be unfamiliar with some of the newer injectables, such as platelet-rich plasma (PRP), mesenchymal stem cells (MSCs), or viscosupplementation. Therefore, a concise summary of each of these is provided. For a more thorough review, faculty can refer to the literature review on injectable biologics and their supporting evidence by Kubrova et al. [8].

PRP is created after a blood sample is drawn from a patient and subsequently centrifuged to separate the platelets and plasma from other components [9]. The solution is then injected into the patient’s target painful area, such as an injured tendon, with the primary goal of accelerating the healing process [9]. A recently published 2023 systematic review and meta-analysis of ultrasound-guided PRP injections for tendinopathies showed that patients suffering from carpal tunnel syndrome experienced a reduction in visual analog scale scores and Boston Carpal Tunnel Questionnaire severity scores compared to controls [10]. However, outcomes for patients suffering from lateral epicondylitis, plantar fasciitis, or Achilles/rotator cuff/patellar tendinopathies were comparable to those observed in the control groups [10].

Stem cells are “undifferentiated cells capable of perpetual self-renewal and can differentiate into specialized cell types” [10]. MSCs, derived from bone marrow or adipose tissue, can rapidly divide and repair damage [11]. Therefore, MSCs are currently being investigated for a variety of conditions, such as discogenic back pain, spinal cord injury, trigeminal neuralgia, diabetic neuropathy, osteoarthritis, musculoskeletal disease, and neuropathic pain conditions [11].

Viscosupplementation involves the injection of a gel-like substance, such as hyaluronic acid, into a joint to reduce pain or swelling [12]. The goal is to provide cushioning and support to an area where natural cartilage may have deteriorated, as seen in patients with osteoarthritis. Interestingly, while these injections have been widely used for conditions such as knee osteoarthritis, a 2023 systematic review and meta-analysis showed superior outcomes with PRP or bone marrow aspirate concentrate (BMAC) compared to hyaluronic acid [13]. Currently, there are five hyaluronates approved by the Food and Drug Administration (FDA): Hyalgan, Supartz, Synvisc, Euflexxa, and Orthovisc [14].

### Minimally invasive procedures

Minimally invasive procedures, typically performed via small incisions or with specialized instruments, include treatments, such as radiofrequency denervation, cryoneurolysis, neuromodulation (i.e., spinal cord stimulation, dorsal root ganglion stimulation, peripheral nerve stimulation, etc.), intrathecal drug delivery, and minimally invasive lumbar decompression (MILD), among others. Each of these procedures has its respective indications and contraindications, as well as associated risks and benefits.

Radiofrequency denervation or cryoneurolysis, in the simplest terms, involves using thermal energy (hot vs cold) to temporarily damage or destroy nerves. Radiofrequency denervation can be utilized for mechanical pain arising from the medial branch nerves of the spine (cervical, thoracic, or lumbar) and is also employed for various osteoarthritic joint pains (i.e., knee, hip, and shoulder). These procedures provide temporary relief, as the nerves regenerate over time and the pain ultimately returns.

Neuromodulation is a broad category of interventional procedures that utilize electricity to interfere with nerve signaling/firing to provide pain relief. Many neuromodulation targets exist, including the dorsal root ganglion, the spinal cord, peripheral nerves, and more. The risks vs benefits and indications vs contraindications for these various device implants depend on the specific nerve target. It is important to note that with numerous neuromodulation devices, patients are allowed a trial period in which a temporary version of the device is implanted. During the trial, patients are encouraged to perform routine activities while documenting their degree of pain and functioning. If the patient experiences a successful trial, they can proceed to permanent device implantation.

### Moderate/highly invasive procedures

Lastly, the most invasive options include “open” procedures, where larger incisions are required, resulting in longer anticipated healing or recovery times. These procedures are completely appropriate in certain patient scenarios but, when possible, should be reserved as a last resort for painful conditions in which alternative treatments have not yet been exhausted and no “red flags” or emergent surgical indications exist.

## Conclusion

There are more options for acute and chronic pain management than ever before, which is fortunate given the increasing prevalence of chronic pain and the ongoing opioid epidemic. A pragmatic approach to helping PA students understand pain management and determine appropriate options for their patients is warranted. This article presented a simple conceptual framework activity for basic pain management that PA faculty can embed within the existing PA program educational curriculum. Furthermore, this approach could also be adopted by other healthcare training programs, such as nurse practitioner or pharmacy education.
